# Hablo Inglés y Español: Cultural Self-Schemas as a Function of Language

**DOI:** 10.3389/fpsyg.2017.00885

**Published:** 2017-05-30

**Authors:** Gloriana Rodríguez-Arauz, Nairán Ramírez-Esparza, Norma Pérez-Brena, Ryan L. Boyd

**Affiliations:** ^1^Department of Psychological Sciences, University of ConnecticutStorrs, CT, United States; ^2^Department of Family and Child Development, Texas State UniversitySan Marcos, TX, United States; ^3^Department of Psychology, University of Texas at AustinAustin, TX, United States

**Keywords:** language, personality, Mexican-Americans, Meaning Extraction Method, self-schemas

## Abstract

Research has demonstrated that bilingual individuals experience a “double personality,” which allows them to shift their self-schemas when they are primed with different language modes. In this study, we examine whether self-schemas change in Mexican-American (*N* = 193) bilinguals living in the U.S. when they provide open-ended personality self-descriptions in both English and Spanish. We used the Meaning Extraction Helper (MEH) software to extract the most salient self-schemas that influence individuals' self-defining process. Following a qualitative-inductive approach, words were extracted from the open-ended essays and organized into semantic clusters, which were analyzed qualitatively and named. The results show that as expected, language primed bilinguals to think about different self-schemas. In Spanish, their Mexican self-schemas were more salient; whereas, in English their U.S. American self-schemas were more salient. Similarities of self-schemas across languages were assessed using a quantitative approach. Language differences and similarities in theme definition and implications for self-identity of bilinguals are discussed.

## Introduction

Would the reader say that these two phrases describe the same person?

Phrase 1: “I'll start off by saying that I am very dedicated to learning and school. I love to learn and getting good grades is a top priority to me.”
Phrase 2: “I am a faithful daughter, girlfriend and friend. I am always side by side with my friends and family, whenever they need me, and I do everything in my power to make them happy.”

The answer is yes. The two phrases belong to a personality description given by a female bilingual Mexican-American student, when responding to the request: “*For the next 15 min, describe your personality”* in two different occasions. In the first phrase the student is answering to the request in English and in the second one she is responding in Spanish. These two phrases exemplify how the same person focuses on different aspects of their life depending on the language used to describe their personality. The student is talking about behaviors, attitudes and feelings associated with a specific self-schema. Self-schemas are defined as those constructs that are chronically accessible and that direct an individual to focus on certain aspects of their life. Self-schemas influence how an individual perceives, remembers, and feels about life experiences (Markus, 1977; Cantor, 1990). For example, when the student in the example provided above is describing her personality in English, the most salient schema is achievement; and when the student is describing her personality in Spanish the most active schema is relationships. In this study we explore Mexican-American's self-schemas when describing their personality to an open-ended question in English vs. in Spanish utilizing an alternative method of data collection and analysis, along with a qualitative-inductive approach.

### Language as a prime that triggers different self-views

In the past few decades, research has shown that bilingual individuals shift their personality when language is used as a prime; and that they change their personality in accordance to the personality differences associated to the culture of that language (Ross et al., 2002; Ramírez-Esparza et al., 2006; Chen and Bond, 2010; Chen et al., 2014). For example, Ramírez-Esparza et al. (2006) asked Spanish-English bilinguals of Mexican descent to participate in two language sessions to trigger two language modes. In the Spanish session, the participants were interviewed in Spanish and then completed the Big Five Inventory in Spanish (Benet-Martínez and John, 1998). The same procedure was repeated in the English session and the participants responded to the Big Five Inventory in English (John and Srivastava, 1999). Results demonstrated that bilinguals were more extraverted, agreeable and conscientious in English than in Spanish. Furthermore, these differences were consistent with the personality displayed in the culture associated with each language. That is, U.S. Americans also scored higher than Mexicans in Extraversion, Agreeableness, and Conscientiousness.

Ramírez-Esparza et al. study (2006) demonstrated that bilinguals shift their personality depending on the language mode, and that they switch their personality to match the personality of the two cultures they identify with. However, the results also raised other questions. For example, it was puzzling that personality differences between U.S. Americans and Mexicans were inconsistent with well-known stereotypes about these cultures. Mexicans are polite and kind; they show respect toward others, avoid conflict by emphasizing positive behaviors and deemphasizing negative behaviors. Cultural scientists have used the cultural script *Simpat*í*a* to label this kind of social interaction among Mexicans and Latinos (Triandis et al., 1984; Díaz-Loving and Draguns, 1999; Holloway et al., 2009). However, when Mexicans and bilinguals respond to the questionnaire in Spanish, they saw themselves as less agreeable than U.S. Americans and bilinguals, when responding to the questionnaire in English. What could account for this paradoxical finding? Ramírez-Esparza et al. (2006) hypothesized that bilinguals expressed changes in their cultural values on self-reports, as opposed to changes in their personality. In other words, response to self-reports interacts with cultural values in bilinguals. Since an underlying characteristic of the cultural script Simpatía is modesty, when bilinguals read a question in Spanish, the modesty value is activated. Individuals' need to sound modest leads them to diminish their standing on agreeableness and other traits (see also Ramírez-Esparza et al., 2008).

Besides the discrepancies noted above between self-reports and behavior with Mexicans, self-reports have broader limitations that are worth discussing. First, the usage of these instruments usually follows the “imposed etic” approach. This strategy operates by administering measures that were developed under a particular cultural worldview that is then implicitly superimposed on members of different cultural groups. While this facilitates the comparison of a construct across cultures, it might also leave out other important constructs that are specific to the cultural group being examined (Benet-Martínez and Waller, 1997). Second, another limitation involves the translation of assessment materials. It is often incorrectly assumed that the act of directly translating measures to another cultural group's language makes the construct valid for them. In other words, translation of instruments can be correct from a linguistic point of view, but the constructs themselves might not resonate psychologically with the members of the cultural group being analyzed (van de Vijner and Tanzer, 2004).

In light of these important challenges, it is worth asking what Mexican-American bilinguals have to say, about themselves, using their own words in each language. By using this approach, we can capture language specific domains of self-definition and bypass the methodological cultural biases that result from using self-reports. Ramírez-Esparza et al. (2006, 2008, 2014) and Ramírez-Esparza and García-Sierra (2014) have proposed that to collect and analyze culture- and language-specific domains, it is important to use alternative methods. In our current study, we use one such alternative approach. That is, we allow individuals to spontaneously express their values, thoughts, and feelings associated to their most salient self-schemas when describing their personality.

### Extracting self-schemas using an open-ended approach

Ross et al. (2002) conducted a study in which they randomly assigned a group of bilingual Chinese-Canadians to describe their personality in an open-ended way in two different languages: English and Chinese. Their goal was to test the assumption that language prompts shifts in cultural frames; for example, when asked to describe their personality in Chinese, participants would be inclined to define their personality more frequently in terms of their relationships with others, their cultural background, and their group memberships, with much less focus on themselves and their traits, as opposed to English. They asked two independent coders to rate how much these four themes appeared in the self-descriptions. The authors indeed found that in Chinese, participants mentioned their cultural background more, they had increased references to others, more group membership references and less statements about themselves, as opposed to English.

Although, Ross et al.'s study (2002) used an alternative measure to extract self-schemas, it has some limitations. First, bilingual participants were assigned either to two independent conditions (English language description or Chinese language description). This does not answer the question of a possible shift of self-schemas within the same individual. Also, the authors were set to look for 4 pre-determined self-schemas in the self-descriptions (references to others, cultural background, group memberships and to oneself). This leaves out other self-schemas that naturally emerge and that might fit in the definition of independent and interdependent selves (e.g., focus on individual achievement vs. relational goals). Moreover, the richness of open-ended data is lost if the goal of the researcher is simply to look for the occurrence of a particular self-schema that is only known to him or her. A final limitation has to do with its usage of the traditional method to analyzing open-ended text. In this approach, independent coders are trained to look for particular themes in the text; this requires time and energy to develop complicated coding schemes and it is not exempt of biases (Chung and Pennebaker, 2008).

In this study, we address these gaps by analyzing the open-ended self-descriptions of a sample of Mexican-American bilinguals in two different languages at two different moments in time, to tap into intra-individual variability. The data is analyzed using an innovative text analytic strategy called the Meaning Extraction Method (MEM; e.g., Chung and Pennebaker, 2008; Boyd, 2015; Stanton et al., 2015; Pulverman et al., 2016). This text analytic approach uses factor analyses to observe how groups of content words cluster together. Each cluster can be thought as an underlying self-schema that guides word choice when describing one's personality. These coherent word clusters will suggest latent dimensions, which will be analyzed qualitatively to define and name the factors. This has been called the inductive-qualitative approach (Chung and Pennebaker, 2008). Relevant to this study, self-schemas have been successfully defined with monolinguals U.S. American participants (Chung and Pennebaker, 2008) and in both monolingual U.S. American participants and monolingual Mexican participants (Ramírez-Esparza et al., 2012).

More explicitly, Ramírez-Esparza et al. (2012) collected open-ended personality descriptions in the same way we intend to do in this study, from a sample of 560 English-speaking U.S. Americans and 496 Spanish-speaking Mexicans. The results from the MEM showed that 8 factors for U.S. Americans and 6 factors for Mexicans brought together a group of content words that are psychologically meaningful and coherent. The authors used previous findings with U.S. American participants (i.e., Chung and Pennebaker, 2008) and an inductive-qualitative approach to name the factors and to observe which factors were found only in one culture (i.e., culture-specific self-schemas) and which ones were similar across cultures (i.e., cross-cultural self-schemas). The results from these analyses showed that some factors were comparable across cultures (e.g., Values, Sociability, and Emotionality). Although, emotions in the U.S. Americans were reflected in two separate factors (i.e., Positivity and Negativity), they appeared together as a single emotional factor in the Mexicans (i.e., Emotionality). Other factors were similar, but were labeled differently because U.S. Americans talked more about their daily activities (i.e., Daily Activities) and Mexicans talked about their specific interests (i.e., Hobbies). Two culture-specific self-schemas were found for Mexicans (i.e., Relationships and Simpatía), and three in the U.S. Americans (Fun, Existentialism, and College Experience). We use findings from this particular study to shape our expectations on the kind of self-schemas that can emerge in Mexican-American bilinguals.

### Study overview and expected self-schemas as a function of language mode

In this study we explore Mexican-American's self-schemas when describing their personality to an open-ended question in English vs. in Spanish. In order to achieve this goal, first we use the MEM, and then we rely on both previous studies (i.e., Chung and Pennebaker, 2008; Ramírez-Esparza et al., 2012) and a inductive-qualitative approach to formulate expectations, name the factors and to observe if there are factors that are specific of a language (i.e., language-specific) and if there are factors that are found across languages (i.e., cross-language). We expect that the language mode will prime individuals to shift their self-schemas to match the cultural values associated with that language. When bilinguals describe their personality in English, their U.S. American cultural self-schemas will emerge, whereas when they describe their personality in Spanish, their Mexican cultural self-schemas will be most salient (i.e., Ramírez-Esparza et al., 2006).

A secondary goal of this investigation is to observe if the resulted self-schemas in English are comparable to the self-schemas in Spanish. In order to achieve this goal we use a quantitative approach following guidelines found in Ramírez-Esparza et al. (2012). Essentially, this approach allows us to examine the degree to which dimensions in one language relate with dimensions in the other language. If they relate, this means that the dimensions are meaningful in both languages and are not language-specific. If they do not relate, this means that the dimensions are meaningful in only one language. Using this quantitative approach Ramírez-Esparza found that some dimensions were culture-specific (that is, they were only relevant for Mexicans or U.S. Americans) and other dimensions were cross-cultural (that is, they were relevant for both cultures). We expect that for the bilinguals we will also find some dimensions that are language-specific and others that are relevant across languages.

### The independent and interdependent self-schemas

Bilinguals in the English mode are expected to show a more independent self-construal characterized by themes related to being sociable, and achievement-oriented (Markus and Kitayama, 1991). For example, Ramírez-Esparza et al. (2012) found in the factor Fun words such as *outgoing, fun, hang*, and *girls* (see also Chung and Pennebaker, 2008). Moreover, in the dimension College Experience, the U.S. Americans used words such as *clean, organized, college, intelligent*. Furthermore, we expect that in the English mode, bilinguals will describe themselves in more abstract and global ways, in accordance with the independent self-construal (Markus and Kitayama, 1998). For example, Ramírez-Esparza et al. (2012) and Chung and Pennebaker (2008) reported a dimension that was labeled as Existentialism and included words such as *traits, attitude, hope, understand, personality*.

In contrast, bilinguals in the Spanish mode will reflect themes related to an interdependent self-construal, characterized by an emphasis on relationships and significant others (Markus and Kitayama, 1991; Triandis, 1995). For example, Ramírez-Esparza et al. (2012) found more interdependent themes in the Mexican participants; the Relationships factor included words such as *parents, family, friend, boyfriend* with everyday activities such as *career, university, school, eat*.

### Emotional expression

We expect that bilinguals will show different patterns of emotional expression in their descriptions depending on the language mode. In the English mode, bilinguals will either use negative emotion words or positive emotions words, while in the Spanish language mode, bilinguals will show both positive and negative emotions within a single self-schema. This hypothesis would be consistent with the idea that individuals from interdependent cultures tend to display increased emotional complexity (i.e., co-occurrence of positive and negative emotions), compared to individuals of independent cultures (Spencer-Rodgers et al., 2010; Grossmann et al., 2016). For example, Ramírez-Esparza et al. (2012), reported that for U.S. Americans emotions were separated in either negative self-schemas (e.g., they used words like *upset, angry, mad*) or positive self-schemas (e.g., they used words like *happy, laugh, good*). Conversely, in the Mexicans only one self-schema emerged that contained both negative emotion words (e.g., *hate, sad*) and positive emotion words (e.g., *cheerful, happy*).

### Language-specific self-schemas

We expect that the Simpatía schema will be salient only in the Spanish mode (Triandis et al., 1984; Díaz-Loving and Draguns, 1999; Ramírez-Esparza et al., 2008; Holloway et al., 2009). This would be consistent to what Ramírez-Esparza et al. (2012) reported: Mexicans used words like *kind, noble, affectionate, honest*; but U.S. Americans did not use words that reflect Simpatía.

Other self-schemas that were found in Ramírez-Esparza et al. (2012) that were comparable across cultures but slightly different were Daily Activities for the U.S. Americans and included words as *play, sports, years, school, day, stay*, whereas the Hobbies for the Mexicans included words such as *music, read, travel, movies, parties*. Therefore, it is possible that bilinguals in the English mode they will talk more about their daily activities and in Spanish they will talk more about their hobbies.

### Cross-language self-schemas

Finally, we expect to find dimensions that will emerge both in the English and Spanish mode. This will be consistent with the idea that bilinguals are affected by norms, environment, and institutions of the context they are immersed (e.g., Rentfrow et al., 2008). For example, Ramírez-Esparza et al. (2012), found that Values was a meaningful self-schema for both U.S. Americans and Mexicans. U.S. Americans used words such as *family, life, God, respect;* similarly Mexicans used words such as *learn, work, money, life, God*.

## Methods

### Participants

The initial sample consisted of 310 bilingual participants, which self-identified as bilingual and Mexican-American. Out of these initial sample, 236 completed participation in all phases of the study. Out of these participants, 74 were not included in the study because they completed either the essay in English only (*n* = 44) or in Spanish only (*n* = 30). Participants were additionally excluded on the basis of not identifying as Mexican-American; failure to show an adequate level of Spanish-English bilingualism; and whose self-descriptive essay did not have a total word count of at least 50 words, which suggests that they had not taken the 15-min self-description writing task seriously. The final sample comprised of 193 bilingual participants (44 men and 149 women) with a mean age of 23.43 (*SD* = 4.92) years old. Participant's self-identified socioeconomic status was 62.10% working to lower-middle class, 27.40% middle-class, and 9.50% upper-middle to upper class. Per our sampling, 100% of participants self-identified as bicultural (Mexican living in the US or born in the US from Mexican descent).

Seventy-four percent (*n* = 142) of participants indicated that they have been born in the United States, while the remaining 26.40% (*n* = 51) indicated Mexico as their country of birth. All participants indicated a level of confidence of at more than 90% regarding hearing, speaking and reading in both English and Spanish.

### General procedure

Participants resided in various parts of the United States and were recruited to participate via online postings in different outlets, such as Latino and/or Chicano studies centers, flyers, and by sending emails to a wide audience that self-identified as Latino within a university located in southwestern U.S. Participants were offered $20 in gift cards from a popular online store in exchange for participation. Prospective participants were sent a link to an online bilingualism assessment and a demographic questionnaire. This was done as a first step, to ensure participant's eligibility in terms of language proficiency. Participants were included in the study if their self-identity matched inclusion criteria and if they indicated at least 75% confidence in reading, hearing and speaking in both languages. As a second step, eligible participants were sent a link to either one of the two parallel online questionnaires that were created in both English or Spanish to establish the language modes. This was done in a counterbalanced way (e.g., participant 1 was sent the survey in English, while participant 2 was sent the survey in Spanish).

Thirdly, participants that completed the first survey waited for approximately a week until they received the second survey. The language of the second survey depended on the language of the first administration.

After reading a description of the study and giving their consent, participants were asked to describe their personality for 15 min. The instructions for the English personality description were as follows:

“*Personality has been defined as an individual's characteristic traits, behaviors, and attitudes. For the next 15 min, describe your personality.”*

Both online questions had the same physical appearance, the only difference was the language in which they were presented. A 15-min timer was presented on the writing web page, which included a large blank text field for typing. Each of the self-descriptions was formatted as a single plain text file. A spell-check was performed for each text file. The mean word count was 186.30 (*SD* = 111.27) for the English text files and 157.67 (*SD* = 92.00) for the Spanish text files.

### Analytic strategy

#### The meaning extraction method (MEM)

To determine self-schemas in either English or Spanish, the MEM was conducted on the writing samples. As a first step, the most frequently used content words were identified. Frequency counts were taken of all words in the self-descriptions, excluding closed-class words (e.g., articles, auxiliary verbs, prepositions, pronouns) and words that had to be converted to their root form due to tense or plurals (e.g., *ran* had to be converted under *run*) using a computerized word counter, the Meaning Extraction Helper (MEH; Boyd, 2015). The MEH was run separately for the essays in English and the essays in Spanish.

The most frequently used content words that appeared in at least 3.5% of all text files were selected. A total of 233 and 144 content words from English and Spanish text files, respectively, were kept for further analyses. The Spanish essays had less content words given that the amount of closed-class words and conversions that had to be used in this language was higher (for example, the infinitive verb learn (*aprender*) had at least two different tenses that could be converted to it: learned (*aprend*í; past tense), I have learned (*aprendido*; participle form).

The MEH provides a binary output (i.e., “one-hot” encoded) file for each essay, in which particular words are coded for presence (coded as 1) or absence (coded as 0). The final data summary, then, can be thought of as a (Number of Self-Descriptions x Number of Words) matrix, with each entry referring to the presence or absence of each term within each essay. A total of two matrices were set up to accomplish the main goal of this study: 193 (Participants' Self-Descriptions in English) × 233 (English Content Words) matrix, and a 193 (Participants' Self-Descriptions in Spanish) × 144 (Spanish Content Words) matrix. As a final step, a principal components analysis with varimax rotation was conducted with these binary files.

## Results

The main goals of this study were to define self-schemas in bilinguals according to the language mode and to explore cross-language and language-specific self-schemas using a qualitative approach. A secondary goal was to use a quantitative approach to observe the degree that self-schemas in one language are comparable to self-schemas in the other language. Next, the most frequent words used in both languages are provided. Then, self-schemas derived using the MEM are defined for both languages.

### Word frequencies in the English and in Spanish language mode

Table 1 shows the Top 40 most frequently used content words by the bilinguals in both English and Spanish. Note that many of the Top 10 most frequent words in the English text files were also within the Top 10 most frequent words in the Spanish text files (i.e., *time, family, work, life, friend, and learn*). Interestingly, in the essays in English, bilinguals used a larger amount of words about being outgoing and sociable (e.g., *social, outgoing, meet, friendly, quiet, shy*); whereas in Spanish, they used more words related to their roles (e.g., *education, university, work, woman*), hobbies (e.g., *read, travel, study*) and bicultural experience (*Spanish, Mexico, culture*).

**Table 1:** Most frequently used words in English and Spanish text files.

	**Words in the English text files**	**Percentage in texts**	**Words in the Spanish text files**	**Percentage in texts**
1	Time	54.92	VIVIR/Live	42.49
2	Love	46.63	AMIGOS/Friends	47.67
3	Family	39.90	TIEMPO/Time	40.93
4	Work	36.79	FAMILIA/Family	47.15
5	Life	40.93	TRABAJO/Work	34.20
6	Enjoy	33.68	AYUDA/Help	34.72
7	Friend	35.23	SENTIR/Feel	26.94
8	Learn	26.94	HABLAR/Talk	24.87
9	Hard	25.91	MÉXICO/Mexico	18.65
10	School	19.69	APRENDER/Learn	23.32
11	Care	24.35	BUENO/Good	26.42
12	Give	22.28	PADRES/Parents	22.28
13	See	16.58	PROBLEMAS/Problems	19.17
14	Happy	20.73	CULTURA/Culture	17.10
15	Parent	16.58	IMPORTANTE/Important	21.76
16	Shy	21.76	FELIZ/Happy	18.65
17	Goal	20.21	TÍMIDA/Shy	19.17
18	Positive	16.06	SOCIABLE/Sociable	16.58
19	Attitude	18.13	ESCUELA/School	14.51
20	Talk	17.62	MUJER/Woman	14.51
21	Social	16.58	MUNDO/World	13.99
22	Important	15.03	AMABLE/Kind	16.06
23	Strong	14.51	TRABAJAR/To Work	13.47
24	Speak	15.54	LEER/Read	16.06
25	Quiet	15.03	META/Goal	15.54
26	Change	13.99	FÁCIL/Easy	14.51
27	Culture	12.44	ESPAÑOL/Spanish	9.33
28	Meet	13.99	ESTUDIAR/Study	13.47
29	Friendly	13.99	EDUCACIÓN/Education	10.88
30	Outgoing	17.10	RESPONSIBLE/Responsible	13.99
31	Honest	15.03	SOLA/Alone	11.92
32	Living	12.95	DIFÍCIL/Difficult	11.40
33	Laugh	10.36	UNIVERSIDAD/University	11.92
34	Open	13.99	ESPECIAL/Special	11.92
35	Grow	11.40	INTELIGENTE/Intelligent	12.44
36	Place	10.36	ACTITUD/Attitude	10.36
37	Mexican	9.84	ESTUDIO/To study	10.88
38	Responsible	12.95	HONESTA/Honest	11.92
39	Funny	11.40	VIAJAR/Travel	10.88
40	Respectful	10.88	FUERTE/Strong	10.88

A principal components extraction with varimax rotation was performed independently on the words from the English self-descriptions and on the words from the Spanish self-descriptions. Factors were extracted, based on a scree of Eigenvalues for the principal components (Cattell, 1966). Factors at the elbow bend that had Eigenvalues above 1, and provided a significant increment to the cumulative percent variance were selected. As in Chung and Pennebaker (2008) and Ramírez-Esparza et al. (2012) studies, words with factor loadings ≥ |0.20| or higher were retained.

### Self-schemas in the English language mode

The first 6 factors accounted for 14.5% of the total variance. As can be seen in Table 2, 6 factors bring together a group of content words that are psychologically meaningful and coherent. Four factors were comparable to those found by Ramírez-Esparza et al. (2012) in monolingual U.S. Americans: Factor 1 (College Experience), which included words such as *class, university, health, job, and competitive*; Factor 2 (Values) which included words such as *belief, money, country, pride*; Factor 4 (Existentialism) includes words such as *important, future, responsible, dream*; and Factor 6 (Sociability) which includes words such as *friendly, social, group, introvert*.

**Table 2:** Self-schemas from self-descriptions in English: a varimax-rotated principal components analysis.

**Self-schema factors in the English mode**											
**Factor 1: College Experience**		**Factor 2: Values**		**Factor 3: Bicultural Identity**		**Factor 4: Existentialism**		**Factor 5: Daily Difficulties**		**Factor 6: Sociability**	
University	0.61	Support	0.62	Born	0.65	Member	0.48	Quality	0.40	Introvert	0.44
Finish	0.55	Connect	0.55	Mexican	0.55	Role	0.48	Realize	0.40	Critical	0.39
Mother	0.54	Belief	0.50	American	0.55	Joke	0.44	Patient	0.37	Focus	0.39
Health	0.48	Confident	0.40	Culture	0.54	Sister	0.43	Afraid	0.37	Gathering	0.39
Job	0.47	Money	0.39	Graduate	0.44	Important	0.41	Organize	0.37	Quiet	0.38
School	0.46	Country	0.38	Dance	0.43	Strong	0.38	Appreciate	0.34	Idea	0.36
Father	0.46	Woman	0.37	Mexico	0.38	Future	0.35	Decision	0.34	Social	0.36
College	0.43	Relate	0.37	Student	0.38	Outspoken	0.35	Perfectionist	0.33	Ability	0.33
Degree	0.40	Goal	0.35	Education	0.36	Hardwork	0.34	Time	0.32	Dedicate	0.32
Movie	0.39	Experience	0.35	Living	0.35	Responsible	0.33	Pretty	0.31	Group	0.32
Texas	0.38	Challenge	0.34	Grow	0.34	Listener	0.29	Give	0.30	Anxious	0.28
Choose	0.35	Food	0.34	Share	0.33	Public	0.29	Power	0.30	Reflect	0.28
Attention	0.34	Failure	0.33	Reliable	0.32	Dream	0.29	Walk	0.29	Curious	0.27
Work	0.31	Change	0.32	Busy	0.32	Smart	0.29	Stop	0.28	Environment	0.26
Extreme	0.31	Volunteer	0.32	Bring	0.31	Passionate	0.28	Hate	0.28	Comfortable	0.26
Girl	0.31	Pride	0.32	Child	0.31	Understand	0.27	Strive	0.27	Activity	0.26
Age	0.31	Life	0.30	Family	0.29	Serious	0.27	Timid	0.26	Shy	0.25
Class	0.30	Independent	0.30	Interest	0.28	Talk	0.26	Action	0.25	Stubborn	0.24
Hurt	0.30	Humble	0.30	Love	0.27	Kid	0.25	Hard	0.24	Friendly	0.22
Cry	0.29	Procrastinate	0.30	Language	0.27	Long	0.25	Fight	0.22	Impulsive	0.22
Reason	0.27	Stranger	0.29	Eat	0.24	Friend	0.25	Stress	0.22	Develop	0.21
Sensitive	0.27	Plan	0.29	Guess	0.23	Funny	0.25	Affect	0.20	Life	−0.28
Start	0.25	Conversation	0.27	Parent	0.22	Career	0.23	Music	−0.29	Active	−0.22
Play	0.22	Great	0.27	Approach	0.21	Leader	0.23	Hope	−0.26	Fault	−0.22
Competitive	0.21	Struggle	0.27	Enjoy	0.21	Close	0.22	Watch	−0.25	Unique	−0.21
		Seek	0.26	Opportunity	0.21	Trust	0.21	Positive	−0.22		
		Place	0.26	Spontaneous	0.21			Personal	−0.21		
		Problem	0.26	Book	0.21						
		Learn	0.25	Study	0.20						
		Task	0.25	Sure	0.20						
		See	0.24	Young	0.20						
		Difficult	0.21	Emotion	−0.23						
		Knowledge	0.21	Motivate	−0.21						
		Behavior	0.20								

Two factors were unique to the bilinguals and were not comparable to the U.S. American monolinguals from Ramírez-Esparza et al. (2012): Factor 3 (Bicultural Identity), which includes words such as *culture, Mexican, American, language;* and Factor 5 (Daily difficulties) which includes words such as *hard, stress, afraid*, and words such as *organize, strive, time, perfectionist, stop*.

### Self-schemas in the Spanish language mode

The first 5 factors accounted for 14% of the total variance. As shown in Table 3, the 5 factor solution yielded a set of coherent dimensions. Five factors were comparable to those found by Ramírez-Esparza et al. (2012) in monolingual Mexicans: Factor 1 (Relationships) includes words related to relationships, and everyday activities, such as *father, daughter, mother, siblings, education, work, write student, goal*; Factor 3 (Values) includes words such as *improve, change, open, understand, friends, chat, psychology*; Factor 4 (Simpatía), which included words such as *honest, affectionate, friendly, sincere, humble*; and Factor 5 (Hobbies) which included words such as *music, read, dance, study, travel*.

**Table 3:** Self-schemas from self-descriptions in Spanish: a varimax-rotated principal components analysis.

**Self-schema factors in the Spanish mode**									
**Factor 1: Relationships**		**Factor 2: Bicultural Identity**		**Factor 3: Values**		**Factor 4: Simpat**í**a**		**Factor 5: Hobbies**	
PAPÁ/Father	0.51	AMERICANA/American	0.72	LUGARES/Places	0.58	HONESTA/Honest	0.52	MÚSICA/Music	0.55
ORGULLOSA/Proud	0.47	CULTURA/Culture	0.67	NUEVOS/New	0.57	CARIÑOSA/Affectionate	0.44	TEXAS/Texas	0.50
IDIOMA/Language	0.43	ESPAÑOL/Spanish	0.67	DIFÍCIL/Difficult	0.40	DIVERSIÓN/Fun	0.44	PELÍCULAS/Movies	0.46
APRECIO/Esteem	0.39	INGLÉS/English	0.65	MEJORAR/Improve	0.39	ORGANIZADA/Organized	0.43	VIAJAR/Travel	0.41
PEQUEÑA/Little	0.39	MÉXICO/Mexico	0.57	CAMBIAR/Change	0.38	RESPONSABLE/Responsible	0.42	DISFRUTO/Enjoy	0.40
HIJA/Daughter	0.39	HABLAR/Talk	0.44	PENA/Sorrow	0.37	TÍMIDA/Timid	0.40	LEER/Read	0.40
CASA/Home	0.38	PENSAR/Think	0.42	ABIERTA/Open	0.37	AMIGABLE/Friendly	0.38	ESCUCHAR/Listen	0.36
TRABAJAR/Work	0.38	SENTIR/Feel	0.41	BUENO/Good	0.35	CREATIVA/Creative	0.31	ESTUDIO/Studies	0.32
UNIVERSIDAD/University	0.38	VIVIR/Live	0.35	FÁCIL/Easy	0.33	ALEGRE/Cheerful	0.28	BAILAR/Dance	0.31
HERMANOS/Siblings	0.38	IDENTIFICO/Identify	0.33	ATENCIÓN/Attention	0.32	INTELIGENTE/Intelligent	0.27	IMPORTANTE/Important	0.28
PASIÓN/Passion	0.36	MENTE/Mind	0.32	ENTENDER/Understand	0.31	SINCERA/Sincere	0.27	JUGAR/Play	0.28
MADRE/Mother	0.36	PAÍSES/Countries	0.28	ESPECIAL/Special	0.31	LÍDER/Leader	0.27	ACTIVIDADES/Activities	0.24
PADRES/Parents	0.36	RAÍCES/Roots	0.26	AMIGOS/Friends	0.28	CONFIABLE/Reliable	0.27	MUJER/Woman	0.24
CORAZÓN/Heart	0.35	RESPETO/Respect	0.24	PLATICAR/Chat	0.27	TRABAJO/Work	0.24	LIBROS/Books	0.23
CUMPLIR/Fulfill	0.35	AMOR/Love	−0.25	LEAL/Loyal	0.26	SOCIABLE/Sociable	0.24	FAMILIA/Family	0.22
ESCRIBIR/Write	0.35	NIÑOS/Children	−0.21	NECESITO/Need	0.24	AMABLE/Kind	0.22	ESTUDIAR/Study	0.21
META/Goal	0.35			PSICOLOGÍA/Psychology	0.22	HUMILDE/Humble	0.22	EJERCICIO/Exercise	0.21
LOGRAR/Achieve	0.34			APRENDER/Learn	0.21	ENERGÍA/Energy	−0.27	NOTICIAS/News	0.21
DIOS/God	0.34					EMOCIONES/Emotions	−0.22	PROBAR/Try	0.21
CARRERA/Career	0.33							ENCANTAN/I love it	0.21
GRANDE/Big	0.33							CARÁCTER/Character	−0.24
PERFECCIONISTA/Perfectionist	0.32							ACTITUD/Attitude	−0.23
EXPERIENCIA/Experience	0.32							POSITIVA/Positive	−0.22
HERMANA/Sister	0.30								
ESCUELA/School	0.30								
RELACIONES/Relationships	0.30								
EDUCACIÓN/Education	0.29								
PÚBLICO/Public	0.29								
ESTUDIANTE/Student	0.28								
AYUDA/Help	0.25								
REALIDAD/Reality	0.22								

One factor was unique to the bilinguals and was not comparable to the Mexican monolinguals from Ramírez-Esparza et al. (2012), but was similar to those found in the English self-descriptions was: Factor 2 (Bicultural Identity) and included words such as *American, Mexico, culture, live, identify, countries, roots*.

### Quantitative approach: testing the degree that self-schemas relate across languages

Even though this paper uses an inductive-qualitative approach to extract and name self-schemas within the essays by language, a secondary goal of this investigation was to use a quantitative approach. This approach was used to better understand the degree to which self-schemas are language-specific (i.e., they are only relevant in that language) or interdependent (i.e., they are relevant in both languages). In order to achieve this goal we used the Linguistic Inquiry Word Count software (LIWC2015; Pennebaker et al., 2015). LIWC2015 counts the words that belong to a particular category of words within text files. A user-defined dictionary directs LIWC2015 as to which words or categories of words to search for. In order to quantify if word usage is comparable across languages, we used what we called the *quantitative* approach (also see Ramírez-Esparza et al., 2012). This approach consisted in using a translation process to be able to relate dimensions from the Spanish language with the English language and vice versa. Specifically we performed the following four steps:

1. LIWC2015 program was used to quantify the percentage of words that belong to each factor that appear in a given essay. In order to do this, a dictionary was created where each word that belonged to a factor comprised a single LIWC2015 category. For example, the dictionary would include a category called College Experience and each word within the category (e.g., university, finish, job, school) would become part of this dictionary “College Experience.” This step was done for each of the resultant schema themes in both languages.
2. Using these dictionaries and LIWC2015, we assessed the percentage of words used in each file that comprised each dimension. Specifically, the English dictionary was run on the English text files and the percentage of words used for each English dimension was assessed for each participant. Likewise, the Spanish dictionary was run on the Spanish text files and the percentage of words used for each Spanish dimension was assessed for each participant.
3. Dictionaries were translated into the other language (i.e., the English dictionary into Spanish, and the Spanish dictionary into English). Then, the translated English dictionary was run on the Spanish text files, and the translated Spanish dictionary was run on the English text files. The purpose of running the translated English dictionary on the Spanish text files was to obtain the percentage of words used by bilinguals in Spanish for each of the English dimensions. Likewise, the purpose of running the translated Spanish dictionary on the English text files was to obtain the percentage of words used by bilinguals in English for each of the Spanish dimensions.
4. In the English essays, the English dimensions (i.e., the percentages obtained from running the English dictionary on the English essays) were correlated with dimensions found in the Spanish essays (i.e., the percentages obtained from running the translated Spanish dictionary on the English text files). Likewise, in the Spanish essays, the Spanish dimensions (i.e., the percentages obtained from running the Spanish dictionary on the Spanish essays) were correlated with English dimensions (i.e., the percentages obtained from running the translated English dictionary on the Spanish sample). These analyses determined the degree to which dimensions were related across cultures. For example, it was expected that the factor Values from the English dimensions, would correlate highly with the translated factor Values of the Spanish dimensions in the English text files.

Table 4 shows the correlations between the English dimensions and the Spanish dimensions. Correlations from English text files are shown in the top half of the table; correlations from the Spanish text files are shown in the bottom half of the table. Only those correlations that have moderate or above effect sizes are bolded (*r* ≥ 0.30; Cohen, 1988). Positive bolded correlations between the factors and the translated versions of the factors indicate that there is a relation across languages or more interdependence. In contrast, weaker correlations indicate that factors are language-specific or more independent; and they are only relevant in that language.

**Table 4:** Correlations between self-schemas and translated dimensions for both the English and Spanish text files.

		**Translated Spanish dimensions**				
**In the English text files**		**Relationships**	**Bicultural Identity**	**Values**	**Simpatía**	**Hobbies**
*English Language Mode Dimensions*	College Experience	**0.42**	0.04	−0.07	0.26	0.12
	Values	0.12	−0.12	0.15	−0.12	0.01
	Bicultural Identity	**0.34**	**0.48**	0.08	−0.07	**0.43**
	Existentialism	0.02	−0.09	0.25	0.26	0.14
	Daily Difficulties	−0.01	−0.08	0.02	0.04	0.18
	Sociability	−0.21	−0.17	−0.08	−0.01	0.03
		**Spanish language mode dimensions**				
**In the Spanish text files**		**Relationships**	**Bicultural Identity**	**Values**	**Simpatía**	**Hobbies**
*Translated English Dimensions*	College Experience	**0.39**	−0.14	0.01	0.20	0.13
	Values	0.28	0.12	0.08	0.05	0.15
	Bicultural Identity	0.22	**0.36**	−0.03	−0.05	**0.36**
	Existentialism	−0.08	0.05	**0.35**	0.27	0.16
	Daily Difficulties	−0.09	−0.07	0.14	0.01	0.10
	Sociability	−0.20	0.03	−0.07	−0.08	0.08

*Correlations from 0.14 |−14| to 0.17|−17| = p < 0.05; correlations from 0.18 |−18| to 0.22|−22| = p < 0.01 correlations from 0.25|−25| and above = p < 0.001. Only those correlations that have moderate or above effect sizes are bolded*.

#### Cross-language self-schemas

We expected that the factors Bicultural Identity and Values would correlate highly across languages for both the English and Spanish text files. This hypothesis was only partially supported. The Bicultural Identity in the English language mode correlated strongly with the translated Spanish Bicultural Identity dimension in the English text files. Likewise, Bicultural Identity in the Spanish language mode correlated strongly with the translated English Bicultural Identity dimension in the Spanish text files. However, the expected correlations between English Values and Spanish Values across text files were not found.

#### Language-specific self-schemas

Language-specific dimensions for the Spanish language mode such as Relationships, Simpatía and Hobbies, were expected to have low correlations with most of the English language mode dimensions in the English text files. Similarly, language-specific for the English language mode, such as College Experience Existentialism, Daily Difficulties, and Sociability, were expected to have low correlations with most of the Spanish language mode dimensions in the Spanish text files. This was largely supported for both the English and Spanish text files; however, a few interesting correlations in both the English and Spanish text files emerged. The factor Relationships correlated strongly with College Experience in both the English and Spanish text files. This indicates that when bilinguals talk about their college experience in the English language mode, they also talk about their relationships. Likewise, when bilinguals talk about their relationships in the Spanish language mode they also talk about their college experience. The same pattern was found for Hobbies and Bicultural Identity in both the English and Spanish text files. This indicates that when bilinguals talk about their bicultural identity in the English language mode they also talk about their hobbies. Likewise, when bilinguals talk about their hobbies in the Spanish language mode they also talk about their bicultural identity.

One correlation emerged in the English text files that was not as strong in the Spanish text files. Specifically when bilinguals talked about their bicultural identity in the English language mode they also talked about their relationships. Additionally, one correlation emerged in the Spanish text files that was not as strong in the English text files. Specifically, Values in the Spanish language mode correlated significantly with Existentialism.

#### Summary

The quantitative approach allowed us quantify the degree that dimensions are independent (or language-specific) or interdependent (or relevant across languages). We can conclude that the factors are for the most part independent. This means that bilinguals self-defining process is influenced by the language mode. In other words, bilinguals change their self-schemas as they alternate between languages. We further discuss how bilinguals alternate their self-schemas to match the ones found in Mexicans and U.S. Americans in the discussion section. Finally, we expected that the dimension Values would be relevant in both languages; however, the quantitative analyses did not support this expectation. We discuss this finding further in the discussion section.

## Discussion

In this study we explored Mexican-American's self-schemas when describing their personality to an open-ended question in English vs. in Spanish. In order to accomplish this goal, we asked Mexican-American bilinguals to describe their personality in English and then in Spanish (counterbalanced). Then we relied on the Meaning Extraction Method (MEM) to extract the most salient and chronically activated self-schemas that comprise individuals' self-defining process. We expected that bilinguals will exhibit a “double personality” when describing their personality; language mode would prime individuals to shift their self-schemas to match the cultural values associated with that language (e.g., Ross et al., 2002; Ramírez-Esparza et al., 2006). In order to formulate our expectations we referenced to the self-schemas extracted using the MEM from U.S. Americans and Mexicans in Ramírez-Esparza et al. (2012) study. Then we used an inductive-qualitative approach to observe cross-language and language-specific dimensions. Finally, we used a quantitative approach to assess the degree that self-schemas are language-specific or relevant across languages. This to further test the degree that language mode affects bilinguals' “double personality.”

Our results demonstrated that in the English mode, the bilinguals' self-schemas were like the U.S. American monolinguals (i.e., College Experience, Values, Existentialism, Sociability) and in the Spanish mode their self-schemas were like the Mexican monolinguals (i.e., Relationships, Values, Simpatía, and Hobbies). Only two self-schemas in the English mode were not present in the U.S. American monolinguals (i.e., Daily Difficulties, and Bicultural Identity) and one self-schema in the Spanish mode was not present in the Mexican monolinguals (i.e., Bicultural Identity). Furthermore, the quantitative approach demonstrated that as expected, one factor was relevant across languages (Bicultural Identity), but one factor was unexpectedly not relevant across languages (Values). Furthermore, the quantitative approach demonstrated that other factors were language-specific for the English language mode (College Experience, Existentialism, Daily Difficulties, and Sociability) and language-specific for the Spanish language mode (Relationships, Simpatía and Hobbies).

### The independent and interdependent cultural self-schemas

We speculated that bilinguals in the English mode would show a more independent self-construal characterized by themes related to being sociable and achievement oriented (Markus and Kitayama, 1991); and they would also describe their personality in more abstract ways (Markus and Kitayama, 1998). In accordance with our expectations, in the factor Sociability the bilinguals used words such as *introvert, gathering, quiet, social, group, shy*. This demonstrates that when bilinguals are in their English mode they think about being sociable, outgoing, proactive and about maintaining interactions with others. This feature is common within an independent self-construal, where interactions with others are driven by a need for individuals to be leaders and assert themselves in front of others (Ramírez-Esparza et al., 2006). This factor is very similar to the ones reported by Ramírez-Esparza et al. (2012) and Chung and Pennebaker (2008) in monolingual U.S. American participants.

Furthermore, the College Experience dimension presents a series of words that are overall oriented toward achievement (e.g., *university, finish, attention, work, competitive*). This theme falls in line with more individualistic ideals, where a person must challenge him or herself and live up to his or her full potential (Markus et al., 2006). This factor resembles closely the Education factor reported by Chung and Pennebaker (2008) and to the College Experience factor reported by Ramírez-Esparza et al. (2012) in monolingual U.S. American participants.

Finally, the Existentialism factor provides additional insights for an independent self. As Markus and Kitayama (1998) noted, through the lens of an independent self, individuals tend to think about themselves in more abstract and global ways. This is reflected by words such as *role, important, strong, public, close, dream, passionate*. Chung and Pennebaker (2008) and Ramírez-Esparza et al. (2012) reported a similar factor in monolingual U.S. American participants.

Conversely, we hypothesized that bilinguals in the Spanish mode would reflect a more interdependent self, by valuing social relationships and connection to others (Markus and Kitayama, 1991). Our results provide support to this hypothesis. On the Relationships dimension, bilinguals mentioned their relationships (i.e., they used words such as *father, siblings, mother, parents, relationships*) in hand in hand fashion with their everyday activities (i.e., they used words such as, *house, work, university, write, goal, achieve, career, school, student*), just like the Mexicans (see Ramírez-Esparza et al., 2012).

### Emotional expression

We expected that bilinguals would show differential patterns of emotional expression according to the language mode. Following Ramírez-Esparza et al. (2012), words denoting negative and positive emotions would emerge in separate dimensions in English, while in Spanish, these words should appear within a single self-schema. In this study no clear positive or negative valenced dimensions emerged. One of the possible reasons why a not clear positive-valenced and/or negative-valenced factor emerged as in Ramírez-Esparza et al. (2012), is because the bilinguals gave priority to describing their thoughts and feelings about being biculturals. In this sample, we find that in both the English mode and the Spanish mode, bilinguals described their experience of being biculturals, which we will discuss more in detail below.

### Language-specific self-schemas

We expected that a Simpatía schema would emerge only when bilinguals describe their personality in the Spanish mode. Our MEM results confirmed this expectation. Importantly, the quantitative approach demonstrated that Simpatía was only relevant in the Spanish language, further supporting the idea that this dimension becomes only relevant when bilinguals are in the Spanish language mode.

The Simpatía dimension mirrored a well-established cultural-script of Mexicans and Latinos (Triandis et al., 1984; Holloway et al., 2009) where a person high on Simpatía is likeable, easygoing, polite, fun to be with, affectionate, and likes to share feelings with others. Indeed, the bilinguals in the Spanish mode, just like the Mexicans, used words such as *honest, responsible, cheerful, friendly, reliable, sincere, humble, kind*. This finding is important, given that previous studies have reported that both Mexicans and bilinguals when answering to self-reports in Spanish, tend to rate themselves as less agreeable than U.S. Americans (Ramírez-Esparza et al., 2006, 2012). However, when Mexicans and bilinguals are asked to describe their personality in Spanish in an open-ended question, the cultural biases that are primed when answering to self-reports are not prevalent. This shows that alternative methods such as factor analyses of meaningful words can be informative about cultural values associated with communality and maintenance of harmony, which underlie Simpatía (Ramírez-Esparza et al., 2008).

We also speculated in line with Ramírez-Esparza et al.'s (2012) findings that bilinguals in the English mode would talk more about their daily activities, whereas bilinguals in the Spanish mode would talk more about their hobbies. Our results provide partial support for this hypothesis. While we did find a separate self-schema related to hobbies in the Spanish mode (i.e., bilinguals used words such as *music, travel, read, dance, play, study, exercise*) we did not find a self-schema related to daily activities in the English mode. Rather, we found a self-schema that we labeled as Daily Difficulties. This factor includes words about everyday activities (e.g., *organize, time, walk, music, stop, give, action*) and words that have a negative connotation (e.g., *afraid, hate, timid, hard, stress*). It is worth noting, that this factor also contained words pertaining to being conscientious and diligent (e.g., *organize, perfectionist, power, strive, fight*). The Daily Difficulties dimension could be a reflection of a well-established individualistic, cultural ideology present in U.S. Americans: The Protestant Work Ethic. This philosophical system underlies individualism in the United States (Weber, 1958) and proposes that individuals are, above all, capable to work autonomously toward their life goals. Under this lens, hard work, self-discipline and the exercise of personal responsibility are the key to success in life (Feather, 1984). Thus, it is possible that in the English mode, the Protestant Work Ethic is activated and the bilinguals think about the activities they have to do in order to accomplish their goals, including being diligent and conscientious.

### Cross-language self-schemas

Finally, we expected to find dimensions that will emerge both in the English and Spanish mode. This will be consistent with the idea that the bilinguals will be affected by norms, the environment, and institutions of the context they are immersed (Rentfrow et al., 2008). Accordingly, we found two factors to be similar across languages: Values and Bicultural Identity. However, the quantitative approach confirmed that only bicultural identity was relevant in both languages, but Values was not. This latter finding can be better understood if one analyses closely the words used in each of the Values dimensions.

Although, bilinguals in the English and Spanish mode express their values as a salient dimension, there are some apparent differences depending on the language mode they were using. For example, in the English language mode, the factor Values seemed to reflect independence (in words such as *support, connect, money, goal, confident, independent, plan, seek, see)*, and an effort to improve oneself (in words such as *confident, relate, challenge, failure, change, volunteer, learn, knowledge)* On Table 5 is an example of an essay that scored high on Factor 2 (Values) in the English mode. The independent focus that transpires in this essay can be explained in terms of an analytic thinking style (Nisbett et al., 2001). This style is characterized by a cognitive focus on an object that occupies a given context; the object itself is the main source of interest for the perceiver, regardless of the environment that the object is embedded in. In the sample essay, we get the sense that the writer situates herself as the main focus of the narrative. The text references someone that is agentic and dominates her context.

**Table 5:** Example essay with high score in Values factor in English.

*I am a go-getter and a natural leader, I don't take no for an answer, and if I get a no I figure out a way to get a yes plus a little bit more. I carry myself in a very quirky way, I tend to disprove many stereotypes and use those experiences to teach people who seek to impose stereotypes on people. I am a scholar by heart, but find great enrichment in the lives of those who are not academically trained and are taught by life. I am very versatile in getting along with people and talking to different people. I am independent, and I can figure out things on my own or create a solution from a problem. As a result I love when things are challenging and can use my hands to fix a problem. I am optimistic and while I can be consumed in a setback for a few hours things always seem to fall into place. I am passionate and extremely courageous to a point where at times I put myself in problematic and dangerous situations. I am a short-tempered person impulsive and impatient. I am consumed with pride, which causes me to keep my friends circles very small and private. I have been told I am a threat or intimidating but usually end up gaining much respect and friendship from those who feel that way. I have issues empathizing with the emotions of female peers and have always had a difficult time maintaining friendships with Latina women my age*.

In contrast, the factor Values in the Spanish mode also seemed to reflect value for self-improvement (in words such as *improve, change, learn, understand*); rather than showing a value for independence, it shows a value for interdependence or connectedness (in words such as *new, places, open, friends, chat, loyal*). On Table 6 is an example of an essay (translated to English) that scored high on Values factor in the Spanish mode. The interdependent focus that transpires in this essay can also be explained as a holistic thinking style (Nisbett et al., 2001). Rather than seeing the objects as distinct, separate entities on a given context, holistic thinking is characterized by a cognitive focus on the context where an object is embedded. Furthermore, holistic thinking style focuses on the relationships that objects establish (Nisbett et al., 2001). In this example essay, we get a sense that the writer positions herself in function of her context; she is in search of strengthening relationships with others.

**Table 6:** Example essay with high score in Values factor in Spanish translated into English.

*I am a quiet person who does not talk much at first but after listening for a moment, can establish conversations about any topic. I like intelligent people, and I consider myself to be fairly intelligent. I am also creative, so sometimes I tend to be melancholic or a little distracted. I like to travel and take pictures. I like to spend time reading or enjoying a landscape without feeling rushed, but is not something I can do very often. I like doing things for others, particularly connect people with each other. I like to refer people to others who can be of help to them. I'm a little picky, I like what I like and the rest I definitely do not like. I like to learn new things and I would love to learn to play the piano. I would also like to speak many languages. At the time, I defend myself in two languages but I can read and write a little in other two. I consider myself a compassionate person who always tries to be better every day. I'm introspective and very sentimental. I like to love and be loved. Sometimes I am very on top of others, I'm always stealing kisses or hugs or seeking affection of any kind from almost anyone. Among my flaws they are that I am very sloppy. I always start projects and it takes me thousands of hours to complete them. Or then I do not start projects that I want to do because I do not allow myself the time and discipline to do so. I think I'm a pretty open to accept new things or people with different thoughts, but I'm very reserved with my true friendships. My friends always tell me that I will listen to them and that I will always give them wise advice, or at least make them feel supported. However, my pace of life I makes it difficult for me to make time to be with my friends longer than I do. Although, I have no problems making friends (my husband tells me that I don't know no stranger), it is not easy for me to find a true “soul mate” other than my husband. With him I have a truly special relationship. We respect and love each other. We enjoy our company and I am happy holding his hand. With my family I am a little harsh, because I would like that they'd do things differently, but in general, we have a good relationship. They live in Mexico and therefore we have a close relationship, but still, it works*.

In summary, the Values factor in both languages talks about what is important for that particular person, but it is interesting that the expression of these values is driven by the nuances directed by either the independent or the interdependent self-construal. Furthermore, results can also be explained by both analytic and holistic thinking styles, which have been found to be related to independent and interdependent social orientations (Varnum et al., 2010).

Finally, it is worth noting that both English and Spanish writing samples showed a factor related to one's show bicultural identity. However, biculturalism in English is more referred toward a student identity (e.g., *graduate, student, education, reliable, busy*) while in Spanish, the words refer more toward a general introspection/appreciation of the bicultural identity (e.g., *think, feel, identify, roots, mind, love, respect*). What is interesting about this dimension is how the bicultural identity is salient, regardless of language. The fact that themes are equally salient and appear in both English and Spanish can signal a bicultural identity that is fully integrated into the experience of the self, enriching it and making it stronger. The quantitative approach seemed also to support this saliency; given the significant relation this factor had with its translated version in both English and Spanish text files.

Having a bicultural identity that is salient and integrated within one's experience has been operationalized as Bicultural Identity Integration construct (BII) (Benet-Martínez and Haritatos, 2005). This construct refers to the capacity of an individual to integrate two different cultural experiences and see them as complementing parts of the self, rather than parts that are in conflict or opposition (Benet-Martínez and Haritatos, 2005). It has been reported that individuals high in BII show advantages in terms of their subjective well-being. For example, Chen et al. (2008) gathered data on the BII levels and well-being of undergraduates in a Hong Kong university that came from mainland China. The authors reported that those students with higher levels of BII showed higher levels of psychological adjustment (composite measure of indicators of self-esteem, life satisfaction, anxiety, depression and loneliness) compared to those with lower levels of BII. These effects were detected above and beyond the influence of demographic characteristics and personality factors like neuroticism levels. More recently, Schwartz et al. (2015) explored the longitudinal association between BII and levels of psychological adjustment and family functioning in 9th grade Hispanic adolescents that had recently migrated to the United States. Data on BII levels was gathered across five different time points for the adolescents. In another study, data was gathered on various indicators of psychological well-being and family functioning. The authors report that two trends were detected across the 5 initial time points: high and low levels of BII. At the sixth time of measurement, the adolescents with high levels of BII also tended to show higher levels of self-esteem, optimism, and more fulfilling family relationships, compared to those lower in BII (Schwartz et al., 2015). Hence, there is support for the idea that a salient and strong bicultural identity might signal full integration of this identity within the self. In turn, a full integration is associated with psychological advantages in terms of subjective well-being. This is an important and interesting question that should be addressed in future studies. Specifically, it is important to observe how dimensions of bicultural identity that emerge using an open-ended approach relate to self-reports of BII and other well-being variables.

### Future research

An important line of research that needs further exploration is the relationship of the themes explored here and other variables related to individual success such as psychological well-being, academic achievement and community engagement. As we outlined before, previous studies provide support for the idea that individuals that have incorporated their bicultural identity tend to feel better psychologically (e.g., Chen et al., 2008). Other evidence points out to the potential association between this “double personality” and academic performance. Brannon et al. (2015) reported evidence that when African Americans engaged in cultural activities relevant to their identity, they are more prone to activate their interdependent self-schema, which in turn, appeared related to enhanced academic performance. Naumann et al. (2017) found that Mexican Americans might shape their support for certain social causes depending on their identification with either their Euro-American identity or their Mexican identity. It would be interesting to find evidence of the linkage between the independent and interdependent self of Mexican Americans and these indicators of individual success.

Another line of research worth pursuing is to test if the noted differences in independent vs. interdependent self-construal can cause differences in thinking or cognitive styles. Previous evidence points out that social orientations (independent vs. interdependent) and cognitive styles (analytic vs. holistic) are distinct constructs that have a positive relationship (Grossmann and Na, 2014).

A final line of future research that is worth exploring has to do with complementing these findings with behavioral data. Our study shows that there are certainly differences regarding how bilinguals describe themselves according to language, but what about real life? How do bilinguals switch frames in real life situations? Previous studies have been applied successfully to gather behavioral data on Mexican and U.S. American participants; interesting comparisons have been made on how sociable members of this culture are, as opposed to how they behave in the real world (Ramírez-Esparza et al., 2009). Behavioral data on how bilinguals behave and frame-switch according to cultural cues that occur in a naturalistic environment would add significant richness to the findings that we have exposed here. In addition, future research should also follow a multimethod approach. For example, studies should test if the dimensions reported here converge with reports from outside observers, along with self-reported data. This design has been used successfully in the past to replicate the structure of self-report measures such as the NEO-PI (McCrae et al., 2005).

## Conclusions and implications

Overall, these findings provide support for the claim that bilinguals do exhibit a “double personality”: distinct themes related to independence and interdependence can be found in bilingual Mexican-American individuals, which stem from equally salient self-schemas. When language primes occur, individuals shift to the cultural values more associated to the priming language. Using both a qualitative-inductive approach and a quantitative approach, this paper showed relevant self-schemas in English and in Spanish. The factors specific to English relate to college experience, daily difficulties and sociability, among others. Self-schemas specific to Spanish were relationships, hobbies and a desire to maintain harmony and be agreeable. Further exploration is needed to understand how these themes relate to well-being, academic achievement and behaviors happening in the real world. This study demonstrates the importance of asking bilinguals open-ended questions and using text analysis in the quest of understanding the bilingual experience more fully.

It is important to highlight that since in this investigation we collected data online, we were not able to control for the physical context where the bilinguals provided their self-descriptions. Therefore, we cannot tell if context influenced their thoughts and feelings. However, the fact that bilinguals switched their self-schemas to match the self-schemas of their two cultures (Mexico and the U.S.) indicates that language defeated the cultural context. Hence, language primed the emotions, beliefs, and values associated with that language. For example, it is likely that the bilinguals in the English mode thought about cultural contexts where this language is salient (perhaps at the university) as shown in the dimension College Experience. Likewise, bilinguals in the Spanish mode thought about cultural contexts where this language is salient (perhaps at their parent's home) as shown in the dimension Relationships. These findings suggest that culture and language are, in essence, inseparable (Chen et al., 2014). When individuals acquire different languages they also incorporate the different cultural systems associated with using each language (Chen and Bond, 2010; Chen et al., 2014). Each cultural system encompasses different norms, perceptions and motivations. When individuals switch between languages they are able to access two different, equally salient cultural systems, which allows them to construe different notions of self. Thus, language can act as a primer for diverging and complementary ways of self-perception.

It is also worth mentioning that the self-construals referenced here might reflect the experience of being a member of the Hispanic minority group facing a mainstream U.S. American culture. In other words, these bilinguals define themselves also in relation to major macro-social divisions, which configure their world-view both toward independence and interdependence. Previous evidence supports this idea. Na et al. (2010) found that members of the same culture can differ in their social orientations. For example, working class adults tend to show more interdependence when compared to middle-class adults, who show a more independent social orientation. This also goes in line with current discussions on how measurements of both social orientations and cognitive styles differ at the individual level (Na et al., 2010). More research is needed to explore if variations in self-definitions by language accompany variations in self-definition according to minority status.

These findings can be extended into several new areas that would benefit not only bilingual individuals, but also the communities where they live in and a nation such as the United States, where Hispanics have driven around half of the population growth in recent years (Humes et al., 2011) and given that by recent estimates, 26% of the U.S. American population speaks a language other than English at home (United States Census Bureau, 2015). For instance, triggering different language primes in traditional educational settings can enrich the experience of bilingual students, by making them more understood and appreciated. Students that feel this way would feel more prone to sharing their language and bicultural experience with others, which would further intercultural dialog and respect.

Another major area of applicability is the clinical setting. As therapists and social workers understand how biculturalism induces a double personality experience, they can also feel more empathy toward the complexities of cognitions, emotions and behaviors that make up clients with two or more identities.

In essence, the findings outlined here provide another window to reach out to the experience of bilingual individuals, which in recent times have been the object of questioning and misunderstanding. This is crucial to counteract those voices that call upon building walls to separate, instead of bridges to communicate and bond.

## Ethics statement

This study was carried out in accordance with the recommendations of Institutional Review Boards from the University of Connecticut and Texas State University with written informed consent from all subjects. All subjects gave written informed consent in accordance with the Declaration of Helsinki. The protocol was approved by the Institutional Review Boards from both universities.

## Author contributions

GR and NR contributed with the research idea and data collection. NP contributed with data collection and RB contributed with data analysis.

### Conflict of interest statement

The authors declare that the research was conducted in the absence of any commercial or financial relationships that could be construed as a potential conflict of interest.
